# Effects of the Minimal Extrathyroidal Extension on Early Response Rates after (Adjuvant) Initial Radioactive Iodine Therapy in PTC Patients

**DOI:** 10.3390/cancers12113357

**Published:** 2020-11-13

**Authors:** Freba Ahmaddy, Vera Wenter, Harun Ilhan, Daniel Wacker, Marcus Unterrainer, Thomas Knösel, Peter Bartenstein, Christine Spitzweg, Sebastian Lehner, Andrei Todica

**Affiliations:** 1 Department of Nuclear Medicine, University Hospital, LMU Munich, 81377 Munich, Germany; Freba.Ahmaddy@med.uni-muenchen.de (F.A.); Vera.Wenter@med.uni-muenchen.de (V.W.); Harun.Ilhan@med.uni-muenchen.de (H.I.); Daniel_Wacker@web.de (D.W.); Marcus.Unterrainer@med.uni-muenchen.de (M.U.); Peter.Bartenstein@med.uni-muenchen.de (P.B.); Sebastian.Lehner@med.uni-muenchen.de (S.L.); 2 Institute of Pathology, Ludwig-Maximilians-University of Munich, 81377 Munich, Germany; Thomas.Knoesel@med.uni-muenchen.de; 3 Department of Internal Medicine IV, University Hospital, LMU Munich, 81377 Munich, Germany; Christine.Spitzweg@med.uni-muenchen.de; 4 Ambulatory Healthcare Center Dr. Neumaier & Colleagues, Radiology, Nuclear Medicine, Radiation Therapy, 93053 Regensburg, Germany

**Keywords:** differentiated thyroid cancer, papillary thyroid cancer, minimal extrathyroidal extension, radioactive iodine therapy

## Abstract

**Simple Summary:**

The aim of our retrospective study was to evaluate the impact of minimal extrathyroidal extension on early response rate after (adjuvant) initial radioactive iodine therapy in patients with papillary thyroid cancer (PTC). We found that response rates after radioactive iodine (RAI) therapy in PTC patients were achieved irrespective of minimal extrathyroidal extension (mETE). Nonetheless, the risk of lymph node metastases involvement was significantly higher in the mETE patient group.

**Abstract:**

Background: Extrathyroidal extension of differentiated thyroid cancer is a poor outcome factor but seems to be less significant in minimal extrathyroidal extension (mETE). However, the impact of mETE on response rate after (adjuvant) initial radioactive iodine (RAI) therapy remains unclear. We therefore compared response rates of patients with classical and follicular variants of papillary thyroid cancer (PTC) according to the updated eighth tumor-node-metastasis (TNM) classification to a control group. Methods: 455 patients with T3 (primary tumor > 4 cm) PTC according to the seventh classification who underwent total thyroidectomy followed by RAI therapy were screened. Patients formerly classified as T3 PTC solely due to mETE were reclassified into patients with T1 (primary tumor ≤ 2 cm) or T2 (primary tumor > 2 cm but ≤ 4 cm) +mETE and compared to a control group of T1/T2 −mETE PTC patients. Results: 138/455 patients were reclassified as T1/2 +mETE and compared to 317/455 T1/T2 −mETE control patients. At initial presentation, +mETE patients showed significantly higher rates of cervical lymph node metastases (*p*-value 0.001). Response rates were comparable in both groups (*p*-value n.s.). N1a/N1b-stage (Hazard ratio, HR 0.716; 95% CI 0.536–0.956, *p*-value 0.024) was identified as an independent prognostic factor for lower response rates. Conclusion: Response rates after RAI therapy were comparable in PTC patients irrespective of mETE but with higher rates of lymph node metastases.

## 1. Introduction

The extent of cancer at time of diagnosis is a key factor to assess the chance of successful treatment outcome [1]. In papillary thyroid cancer (PTC), there is general consensus that gross extrathyroidal extension (ETE) affects prognosis [2,3,4]. However, there has been considerable debate over the years regarding the most appropriate treatment for patients with minimal ETE (mETE), defined as extension to perithyroidal soft tissue or sternothyroid muscle [5,6,7,8,9]. Since January 2018, the new eighth edition of the TNM staging system (published in October 2016) has been used to classify patients with differentiated thyroid cancer (DTC) and predict disease mortality. Several substantial modifications were made to the seventh Union for International Cancer Control/American Joint Committee on Cancer (UICC/AJCC) tumor-node-metastasis (TNM) staging system to improve prognostic power, guide best treatment, and change towards the current trend of “personalized medicine” and risk adapted therapy concepts [10,11]. 

As a key change, mETE detected only on histological examination is no longer a determinant of the T-stage within the TNM classification [12]. The former T3 category included any tumor with >4 cm limited to the thyroid gland or tumors of any size with minimal ETE. In the updated eighth AJCC/TNM staging system, DTC with tumor size of ≤4 cm limited to thyroid gland is staged T1/2-disease regardless of the presence of mETE. Considering the initial risk stratification proposed by the 2015 American Thyroid Association (ATA), the presence of mETE alone upstages low-risk patients to the intermediate-risk group [11]. Therefore, the presence of mETE has a direct impact on clinical patient management. 

The impact of mETE on the clinical outcome is still a matter of debate. Some studies suggest that mETE has no impact on the disease-free survival [6,8,13,14], whereas others could not confirm this data [15,16,17] and report a worse outcome in these patients. These studies focused on long-term outcomes. Undoubtedly, long-term outcomes remain the most crucial endpoint, but on a day to day basis, initial presentation of patients influences clinical decisions, and early response to therapy determines the clinical follow-up examinations (e.g., dynamic risk stratification according to the latest ATA guidelines).

Therefore, the aim of the present study was to investigate the initial clinical presentation and the effect on early response rates in PTC patients treated at our institute, which were reclassified as PTC T1/2 with mETE (+mETE) according to the updated eighth TNM staging system and compared to a control group consisting of PTC T1/2 without mETE (−mETE). 

## 2. Results

### 2.1. Group Analysis

#### 2.1.1. Patient Characteristics 

Patient characteristics are summarized in Table 1. At initial presentation, +mETE patients showed significantly higher rates of cervical lymph node (N1a/N1b-stage) metastases (46% in +mETE patients (63/138) versus 29% in −mETE patients (91/317), *p*-value 0.001). Furthermore, higher initial radioactive iodine (RAI) activity was administered in +mETE patients as compared to the control group (4.6 ± 2.1 GBq in +mETE patients versus 3.3 ± 1.0 GBq in −mETE patients, *p*-value 0.001). Regarding histology, +mETE patients had significantly more often classical PTC than the follicular variant of PTC (75% in +mETE patients (103/138) versus 62% in −mETE (197/317), *p* = 0.010).

Both groups showed no significant differences in terms of age (57 ± 16 years in +mETE patients and 55 ± 14 in −mETE patients, *p*-value 0.504), sex (69% female in +mETE patients (95/138) and 74% in −mETE patients (234/317), *p*-value 0.276) and tumor size (14.9 ± 8.4 mm in +mETE patients and 14.4 ± 8.7 mm in −mETE patients, *p*-value 0.551).

#### 2.1.2. Outcome Analysis 

Six to nine months after RAI therapy, the number of patients presenting without detectable Tg (stimulated Tg < 0.5 ng/mL) was comparable in both groups (77% in +mETE patients (106/138) and 81% in −mETE patients (258/317), *p*-value 0.262). Likewise, 97% of +mETE patients (134/138) and 99% of −mETE patients (313/317), *p*-value 0.241) did not present with relevant residual, cervical, or distant pathological uptake (*p*-value 0.387). Therefore, responder rates (combination of stimulated Tg < 0.5 ng/mL, no relevant uptake in the I-131 whole body scan, unremarkable neck ultrasonography) were similar in both groups (76% in +mETE patients (100/138) and 72% in −mETE patients (242/317), *p*-value 0.379). Furthermore, patients showed comparable responder rates irrespective of the histological subtype (*p*-value = 0.908). The outcome of the whole group is shown in Table 1. 

### 2.2. Subgroup Analysis 

#### 2.2.1. N0/Nx-Subgroup Outcome Analysis 

In the N0/Nx-subgroup analysis, 301 patients (75 +mETE patients, 226 −mETE patients) were evaluated. In total, 189/301 (62.7%) patients presented with N0-stage and 112/301 (37.3%) patients with Nx-stage. No significant differences were found between the two groups in terms of Tg responder rates after stimulation (89% in +mETE patients (67/75) and 88% in −mETE patients (199/226), *p*-value 0.764) as well as non-pathological cervical or distant iodine uptake (93% in +mETE patients (70/75) and 94% in −mETE patients (212/226), *p*-value 0.884). Responder rates were identical in both groups (84% in +mETE patients (63/75) and 84% in −mETE patients (190/226), *p*-value 0.988). Findings are presented in Table 2.

#### 2.2.2. N1a/N1b-Subgroup Outcome Analysis

**Figure 1:**
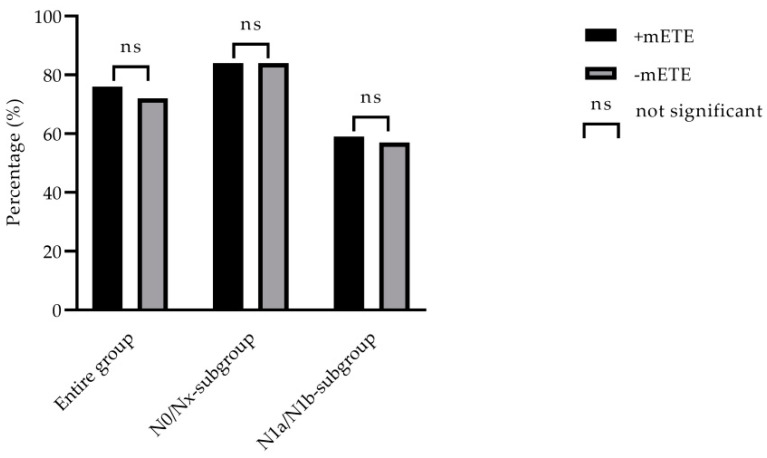
Responder rates (in %) of the entire group and the N-subgroup. No significant differences were found regarding responder rates in the entire group and among N0/Nx- and N1a/N1b-subgroups.

In the N1a/N1b-subgroup analysis, 154 patients were included (63 +mETE patients, 91 −mETE patients). In total, 113/154 (73.4%) patients presented with N1a-stage and 41/154 (26.6%) patients with N1b-stage. In patients with N1a-stage, a mean of 19 ± 13 lymph nodes was removed, and in patients with N1b, 30 ± 20 lymph nodes were removed (*p* = 0.001). There was no significant difference between the two groups in terms of Tg responder rates after stimulation (62% in +mETE patients (39/63) and 65% in −mETE patients (59/91), *p* = 0.710) and relevant iodine uptake in the whole body scan (92% in +mETE patients (58/63) and 87% in −mETE patients (79/91), *p*-value 0.307). Overall, responder rates in this subgroup also were comparable irrespective of mETE (59% in +mETE patients (37/63) and 57% in −mETE patients (52/91), *p*-value 0.845). All data regarding treatment success after follow-up are summarized in Table 2. Irrespective of mETE, in the N1a/N1b-subgroup, subgroup responder rates were significantly lower than in the N0/Nx-subgroup (59% versus 84% in +mETE and 57% versus 84% in −mETE patients, *p*-value 0.001, respectively). In Figure 1, responder rates for the entire group and N0/Nx- and N1a/N1b-subgroups are presented. No differences in responder rates of the entire group as well as among subgroups could be found, irrespective of the presence of mETE. 

Of note, patients with N1a/N1b-status and +mETE received a significantly higher initial radioiodine activity as compared to patients from the −mETE group (6.3 ± 1.7 GBq versus 3.8 ± 1.1 GBq, *p*-value 0.001). In patients with higher administered RAI activity (≥7400 MBq), significantly more patients showed +mETE compared to patients with lower (≤3700 MBq) administered RAI activity (49/57 (86%) patients with high RAI activity versus 89/398 (22%) patients with low RAI activity, *p*-value 0.001). Furthermore, significantly more patients with higher administered RAI activity showed N1-stage (52/57 (91%) patients with high RAI activity versus 102/398 (26%) patients with low RAI activity, *p*-value 0.001). Significantly more patients with higher administered RAI activity showed poorer response rates compared to patients with lower administered RAI activity (25/57 (49%) patients with high RAI activity versus 88/398 (22%) patients with low RAI activity, *p*-value 0.001). 

#### 2.2.3. Uni- and Multivariate Analysis

To analyze possible risk factors for poorer outcomes, we performed a univariate and a multivariate analysis. Of the factors regarding primary presentation of patients, age ≥ 55 years at initial presentation was significantly associated with poorer outcome (Hazard ratio, HR = 7.605, *p* = 0.006), whereas gender (HR = 10.675, *p* = 0.104), histological subtype (HR = 8.123, *p* = 0.746), and the presence of mETE (HR = 9.556, *p* = 0.256) were not associated with poorer outcome. Regarding TNM-staging, N1a/N1b-stage (HR = 7.239, *p* = 0.001) was the only unfavorable prognostic factor for poorer outcome, whereas T-stage > T1 (HR = 8.837, *p* = 0.251) was not associated with lower responder rates. Furthermore, a mean initial I-131-dose of ≥7400 MBq (HR = 14.028, *p* = 0.026) was associated with significantly poorer outcome in the univariate analysis. In the multivariate analysis, N1a/N1b-stage was the only independent unfavorable prognostic factor for treatment success (HR = 0.716, *p* = 0.024). Age ≥ 55 years at initial presentation (HR = 1.003, *p* = 0.405) and mean initial I-131-dose of ≥7400 MBq (HR = 0.915, *p* = 0.672) were not significantly associated with poorer outcome in the multivariate analysis. All risk factors are summarized in Table 3. 

## 3. Discussion

Extrathyroidal extension of thyroid cancer has been recognized as a factor of poor outcome [2,3]. More recent analyses have suggested that mETE is less significant for patient outcome than gross extension [5,6,7,18,19]. The updated eighth edition of the AJCC/TNM staging system removed the subclassification of mETE, resulting in a downstaging of T3 tumors ≤ 4 cm. However, the impact of mETE on treatment success after initial RAI therapy still remains a matter of debate. Although TNM-staging is mainly associated with the disease specific mortality risk, it still influences the clinical patient management. We therefore evaluated the primary presentation and the early treatment response in our patient cohort with or without mETE.

Firstly, we compared +mETE patients to a control group with matched histology, age, sex, and tumor size. Secondly, we subdivided patients in subgroups with N0/Nx- and N1a/N1b- stages to analyze the study group in more detail. These more homogenous subgroups were supposed to improve data quality, as confounding factors could be better analyzed.

Overall, our data suggested that mETE does not affect treatment success. Indeed, analyzing biochemical (Tg-levels) and structural (uptake in WBS and ultrasound) characteristics, comparable responder rates were found in both groups (+mETE vs. −mETE). These findings mirror data by Arora et al. and Kim et al., where the presence of mETE was not an independent predictive factor of recurrence [9,20]. Furthermore, the study by Ito et al. showed that mETE was not associated with recurrence in contrast to gross ETE. Thus, they suggested not using mETE as an indicator for poor prognosis [6]. 

Nevertheless, our data indicate that +mETE patients have a significantly higher risk of presenting with lymph node metastases at initial presentation as compared to −mETE patients. This is in line with the study of Kim et al. who reported that patients with gross or mETE were more likely to have nodal metastases in the central or the lateral neck compartments [20]. Shin et al. showed that there was no significant difference in recurrence between +mETE and −mETE patients; although lymph node metastases were an independent factor for the increased risk of mETE, it did not affect recurrence-free survival [7]. On the contrary, data in a recent meta-analysis from Diker-Cohen et al. indicated that, in patients with N1a/N1b disease, the presence of mETE did further increase the risk of recurrence, yet still within the low risk category [21]. Overall, our analysis identified N1a/N1b-stage as the only risk factor for lower responder rates in the multivariate analysis. Overall, our data indicate that patients with mETE have a higher risk of systemic disease. Therefore, since a prophylactic lymph node neck dissection is not suggested in patients with T1/T2 disease, precise perioperative diagnostic examinations (ultrasound, post-treatment WBS) are crucial in this cohort to exclude lymph node metastases [11].

In our study, response rates were similar in the N0/Nx- and the N1a/N1b-stage subgroup analysis. Nonetheless, it needs to be emphasized that, in this N-stage subgroup, +mETE patients received a significantly higher activity as compared to patients without mETE. Seo et al. compared the outcome after (adjuvant) initial RAI therapy in patients with lymph node metastases and mETE treated with standard activity (3.0 GBq) or a low-dose activity (1.1 GBq) and found no significant differences between both regiments regarding the initial response rate as well as during the follow-up within the first years (mean follow-up time of 45 months) [22]. Therefore, it is very unlikely that the significantly higher treatment activity in our +mETE group might have led to a significant bias in this study. 

Patients with higher administered RAI activity had a worse treatment response compared to patients treated with lower RAI activity. This finding was to be expected, since significantly more patients with lymph node metastases were treated with higher doses. It is well known that patients with lymph node metastases show poorer treatment response [23].

It needs to be highlighted that patients included in this retrospective study come from a historical collective. Therefore, patients received relatively high doses of RAI, which was in line with the German and institutional guidelines at the time of treatment. However, current guidelines trend towards decreasing radioiodine doses, which was supported by the two prospective studies ”Ablation with low-dose radioiodine and thyrotropin alfa in thyroid cancer” (HiLo) and “Strategies of radioiodine ablation in patients with low-risk thyroid cancer” (ESTIMABL) [24,25]. Compared to current guidelines, especially +mETE patients with lymph node metastases were most likely overtreated with RAI. 

As the majority of PTC cases comprise classical and follicular variants of PTC, only these histological subgroups were included in our study [26]. Patients with classical PTC had mETE significantly more often as compared to patients with the follicular variant of PTC. This finding is in line with the study of Yu et al., who also reported significantly higher rates of ETE in classical PTC patients. Response rates in our patient cohort were comparable in both groups. Yu et al. also showed that no differences in outcome could be found between classical and follicular variants of PTC patients [27].

Our study has several limitations. First, there may have been a selection bias because of the retrospective design. Secondly, in our study, patients with mETE were treated with significantly higher radioiodine doses. Higher doses were administered because mETE was considered as a risk factor at the time of inclusion. Furthermore +mETE patients presented more often with lymph node metastases.

Thyroglobulin antibodies are only present in a minority of the patients and therefore could not be included in the analysis in a convincing way. This is indeed a limitation of the study, which cannot be overcome. However, although the recovery is less sensitive than the direct measurement of the antibodies, we are convinced that an undisturbed recovery adds confidence for the validity of the measured thyroglobulin.

Furthermore, primary surgical procedures were performed in different hospitals. Therefore, histological tissue or slides were not available, and only the original pathology reports were reviewed. However, we only included patients with detailed reports indicative for reclassification.

## 4. Materials and Methods 

### 4.1. Study Population

We retrospectively reviewed consecutive patients with pT1-T3 PTC from our institutional data base who underwent total thyroidectomy followed by RAI therapy at the Department of Nuclear Medicine (University Hospital, LMU Munich) between January 2010 and June 2015. Epidemiological and clinical features of these patients were assessed (age at diagnosis, gender, TNM stage, tumor size, presence of ETE, resection margins). Patients initially classified as PTC T3 solely due to mETE (according to the seventh UICC/AJCC TNM staging system) were reclassified according to the updated eighth UICC/AJCC TNM staging system into the subgroup of T1/2 +mETE. These patients were compared to a control group consisting of PTC patients classified as T1/2 −mETE according to former and updated TNM classifications. Pathological reports from the referring hospitals were reviewed and reclassified according to the updated TNM staging system. Since the majority of PTC cases comprise classical and follicular variants of PTC, we only investigated these histological subgroups in our study. Patients with aggressive histological subtypes of PTC, distant metastases diagnosed from clinical examination or imaging (cM1-stage), or unresectable carcinomas or positive resection margins (R1/R2-stage) were excluded. 

For our study, a total of 1140 patients were screened, and 638 patients with PTC were evaluated. A total of 164 patients were retrospectively reclassified as T1/2 with mETE (T1/2 +mETE); 346 T1/2 patients without mETE (T1/2 −mETE) served as a control group. A total of 46 patients remained T3 according to former and updated TNM staging systems and were consequently excluded, and 24 patients were excluded due to aggressive histological subtypes of PTC. A total of 455 patients fulfilled the inclusion criteria. The majority of patients (300/455, 65.9%) showed classical variant of PTC. For subgroup analysis of N0/Nx patients, we defined N0- and Nx-status according to Robinson et al. Patients with T1-stage were staged as N0 after examination of ≥6 lymph nodes and patients with T2 after ≥9 lymph nodes [28]. All other patients were staged Nx.

### 4.2. Ethics Statement

The study was approved by the local ethics committee (Ethics committee of the Medical Faculty, University Hospital, LMU Munich, Munich, Germany, IRB #20-210) and was conducted in accordance with the ethical standards according to the Declaration of Helsinki and according to national and international guidelines. The requirement to obtain informed consent was waived due to the retrospective design of this study.

### 4.3. Treatment

All patients underwent total thyroidectomy with or without lymphadenectomy. Surgery was followed by (adjuvant) initial radioactive iodine therapy [29]. Prior to RAI therapy, patients were stimulated with recombinant human TSH (rhTSH, Thyrogen^®^, Sanofi Genzyme, Cambridge, MA, United States) i. m. on two consecutive days or underwent hormone withdrawal prior to RAI therapy to achieve TSH levels ≥ 30µU/mL according to current guideline recommendations [11]. The administered radioiodine activity depended on the tumor stage as well as on the time of inclusion and ranged from 2.0 to 7.4 GBq I-131 (54–200 mCi). 

### 4.4. Follow-Up and Outcome

Outcome of RAI therapy was assessed six to nine months after initial therapy. The follow up was based on physical examination, neck ultrasound, determination of the level of stimulated thyroglobulin (Tg)-level by two assays (Roche Elecsys^®^ Tg II, Roche Diagnostics GmbH, Mannheim, Germany) with a measuring range of the Roche assay as 0.04–500 ng/mL with a theoretical lower limit of detection of 0.04 ng/mL and a theoretical lower limit of quantitation of 0.1 ng/mL with an error of < 30% or Siemens Immulite with an analytical sensitivity of 0.2 ng/mL and a functional sensitivity of 0.5 ng/mL), and Tg-recovery (Roche Elecsys^®^ Tg II Confirmatory Test) as well as a diagnostic I-131 whole body scintigraphy (WBS), which was performed approximately 72 h after application of 370 MBq I-131 (10 mCi) in hypothyroidism or after administration of rhTSH i.m. on two consecutive days. In case of any pathological finding in the WBS, an additional single-photon-emission computed-tomography (SPECT)/low dose computer-tomography (CT) of the relevant region was performed (most often neck and thorax). Three days after stimulation, Tg and Tg recovery were determined. 

Patients were classified as responders to adjuvant radioiodine therapy if stimulated Tg-levels were lower than 0.5 ng/ml, the thyroid bed was empty or showed a hyperechoic region, and no suspicious lymph nodes were found in the neck ultrasound and if the uptake in the thyroid bed was rated as non-relevant and no pathological uptake was seen outside the thyroid bed according to the WBS. In addition, if a second radioiodine treatment was needed for any given reason, the adjuvant radioiodine therapy was considered as inadequate. 

### 4.5. Subgroup Analysis

At initial presentation, +mETE patients presented significantly more often with lymph node metastases (N1-stage 46% in +mETE patients vs. 29% in −mETE patients) as presented in Table 1. Therefore, we performed a subgroup analysis. We compared patients without local lymph node metastases (N0-stage) or with unknown lymph node status (Nx-stage) to patients with local lymph node metastases in the central (N1a-stage) or the latero-cervical compartment (N1b-stage) to identify possible influence of N-stage regarding response rate.

### 4.6. Statistical Analysis 

All continuous variables (age, tumor size, I-131-dose) were expressed as mean ± standard deviation (SD). Unpaired Student´s t-test was used to compare metric variables (age, tumor size, I-131-dose). Chi-squared test was used to compare categorial variables (gender, N-Stage, uptake in whole body scan, Tg-level after TSH stimulation, TSH stimulation by rhTSH, re-therapy courses). Parameters that showed significant influence on success rate in the univariate analysis were included in the multivariate analysis. Vice versa parameters with *p* values ≥ 0.5 were not included in the multivariate analysis. The multivariate regression model was applied to analyze prognostic factors associated with treatment success. A *p*-value ≤ 0.05 was considered statistically significant. All analyses were performed using SPSS computer software (SPSS Statistics 25, IBM).

## 5. Conclusions

In conclusion, early treatment response in patients with classical or follicular variants of T1/T2 PTC is not significantly affected by the presence of mETE. Despite a higher incidence of lymph node metastases in patients with mETE, prophylactic lymphadenectomy is not suggested in patients in the T1/T2-stage. Therefore, accurate perioperative patient workup, including cervical neck ultrasound and post-treatment WBS, remains crucial.

## Figures and Tables

**Table 1:** Patient characteristics and outcome analysis of the entire group.

*n* = 455 (100%)	T1/2 +mETE *n* = 138 (30%)	T1/2 −mETE *n* = 317 (70%)	*p* Value
**Patient characteristics**			
Age (years)	57 ± 16	55 ± 14	0.054 ^┼^
Female sex–no. (%)	95 (69%)	234 (74%)	0.276 ^╪^
Tumor size (mm)	14.9 ± 8.4	14.4 ± 8.7	0.551 ^┼^
Classical PTC	103 (75%)	197 (62%)	0.010 ^╪^
N1a/N1b-stage–no. (%)	63 (46%)	91 (29%)	0.001 ^╪^
Mean initial RAI activity (GBq)	4.6 ± 2.1	3.3 ± 1.0	0.001 ^┼^
**Outcome analysis**			
Tg-level < 0.5 ng/mL after TSH-stimulation	106 (77%)	258 (81%)	0.262 ^╪^
No pathological uptake in WBS–no. (%)	128 (93%)	291 (92%)	0.729 ^╪^
Unremarkable neck ultrasonography	134 (97%)	313 (99%)	0.241 ^╪^
Responder rates–no. (%)	100 (76%)	242 (72%)	0.379 ^╪^
^╪^ χ2 ^┼^ *t*-test			

mETE, minimal extrathyroidal extension; PTC, papillary thyroid carcinoma; T, tumor; N, nodus; RAI, radioactive iodine; GBq, gigabecquerel; Tg, thyroglobulin; TSH, Thyroid-stimulating hormone; WBS, whole body scan; responder rates: combination of stimulated Tg < 0.5 ng/mL, no relevant uptake in the I-131 whole body scan, unremarkable neck ultrasonography.

**Table 2:** Outcome analysis of the subgroups: N0/Nx and N1a/N1b.

Outcome Parameters	N0/Nx *n* = 301		*p* Value	N1a/1b *n* = 154		*p* Value
	+mETE *n* = 75 (25%)	−mETE *n* = 226 (75%)		+mETE *n* = 63 (41%)	−mETE *n* = 91 (59%)	
**Outcome analysis**						
Tg-level < 0.5 ng/mL after TSH-stimulation	67 (89%)	199 (88%)	0.764 ^╪^	39 (62%)	59 (65%)	0.710 ^╪^
No pathological uptake in WBS–no. (%)	70 (93%)	212 (94%)	0.884 ^╪^	58 (92%)	79 (87%)	0.307 ^╪^
Responder rates–no. (%)	63 (84%)	190 (84%)	0.0.988 ^╪^	37 (59%)	52 (57%)	0.845 ^╪^
^╪^ χ2 ^┼^ *t*-test						

Responder rates: combination of stimulated Tg < 0.5 ng/mL, no relevant uptake in the I-131 whole body scan, unremarkable neck ultrasonography.

**Table 3:** Prognostic risk factors for poorer responder rates (uni-/multivariate analysis).

Covariate	Level	Response Rate (Univariate Analysis)		Response Rate (Multivariate Analysis)	
		HR (95% CI)	*p* Value	HR (95% CI)	*p* Value
Gender	Female Male	Ref 10.675 (6.752–14.577)	0.104		
Age (years)	<55 ≥55	Ref 7.605 (7.220–7.989)	0.006	Ref 1.003 (0.995–1.012)	0.405
Histology	Classical PTC Follicular variant PTC	Ref 8.123 (7.451–8.796)	0.746		
T-stage	T 1>T1	Ref 8.837 (7.081–10.592)	0.251		
N-stage	N0, Nx N1a, N1b	Ref 10.737 (6.917–14.557)	< 0.001	Ref 0.716 (0.536–0.956)	0.024
mETE	−mETE +mETE	Ref 9.556 (6.757–12.355)	0.256		
Mean initial RAI dose (GBq)	≤3.7 ≥7.4	Ref 14.028 (5.044–23.012)	0.026	Ref 0.915 (0.605–1.383)	0.672

T, tumor; N, nodus; mETE, minimal extrathyroidal extension; GBq, gigabecquerel; HR, hazard ratio; CI, confidence interval; RAI, radioactive iodine.
